# Poly[ethyl­enediaminium [di-μ-aqua-(μ_6_-benzene-1,2,4,5-tetra­carboxyl­ato-κ^10^ *O* ^1^,*O* ^1′^:*O* ^2^,*O* ^2′^:*O* ^2′^:*O* ^4^,*O* ^4′^:*O* ^5^:*O* ^5^,*O* ^5′^)dithallium(I)]]

**DOI:** 10.1107/S1600536808040282

**Published:** 2008-12-06

**Authors:** Masoud Rafizadeh, Faranak Manteghi

**Affiliations:** a Faculty of Chemistry, Tarbiat Moallem University, Tehran, Iran

## Abstract

The title compound, {(C_2_H_10_N_2_)[Tl_2_(C_10_H_2_O_8_)(H_2_O)_2_)]}_*n*_, was prepared using (enH_2_)_2_(btc)·2H_2_O and thallium(I) nitrate (en = ethyl­enediamine and btcH_4_ = benzene-1,2,4,5-tetra­carboxylic acid). The enH_2_ cation and btc ligand are each located on an inversion centre. The Tl^I^ atom is seven-coordinated by three btc ligands and two water mol­ecules in an irregular geometry due to the stereochemically active lone pair on the Tl centre. The water mol­ecule and btc ligand are bonded to the Tl atoms in μ- and μ_6_-forms, respectively, leading to a three-dimensional structure. The crystal structure involves O—H⋯O, N—H⋯O and C—H⋯O hydrogen bonds, and also a Tl⋯π inter­action of 3.537 (1) Å.

## Related literature

For general background, see: Akhbari & Morsali (2008 ▶); Day & Luehrs (1988 ▶); Fabelo *et al.* (2005 ▶); Murugavel *et al.* (2000 ▶); Shimoni-Livny *et al.* (1998 ▶). For related structures, see: Li *et al.* (2008 ▶); Rafizadeh *et al.* (2005 ▶, 2007*a* ▶,*b* ▶). For the ligand synthesis, see: Rafizadeh *et al.* (2006 ▶).
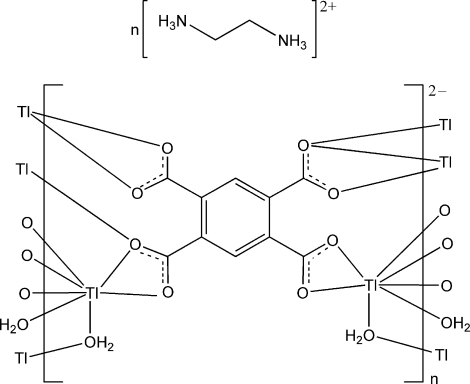


## Experimental

### 

#### Crystal data

(C_2_H_10_N_2_)[Tl_2_(C_10_H_2_O_8_)(H_2_O)_2_)]
*M* *_r_* = 757.01
Monoclinic, 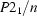

*a* = 9.925 (5) Å
*b* = 7.073 (4) Å
*c* = 11.325 (6) Å
β = 98.397 (10)°
*V* = 786.5 (7) Å^3^
*Z* = 2
Mo *K*α radiation
μ = 20.53 mm^−1^
*T* = 100 (2) K
0.16 × 0.12 × 0.08 mm

#### Data collection

Bruker APEXII CCD diffractometer
Absorption correction: multi-scan (*SADABS*; Bruker, 2001 ▶) *T*_min_ = 0.064, *T*_max_ = 0.201
5272 measured reflections
1787 independent reflections
1487 reflections with *I* > 2σ(*I*)
*R*_int_ = 0.061

#### Refinement

*R*[*F* ^2^ > 2σ(*F* ^2^)] = 0.031
*wR*(*F* ^2^) = 0.070
*S* = 1.00
1787 reflections
107 parameters
H-atom parameters constrained
Δρ_max_ = 2.02 e Å^−3^
Δρ_min_ = −1.80 e Å^−3^

### 

Data collection: *APEX2* (Bruker, 2007 ▶); cell refinement: *SAINT* (Bruker, 2007 ▶); data reduction: *SAINT*; program(s) used to solve structure: *SHELXTL* (Sheldrick, 2008 ▶); program(s) used to refine structure: *SHELXTL*; molecular graphics: *SHELXTL* and *Mercury* (Macrae *et al.*, 2006 ▶); software used to prepare material for publication: *SHELXTL*.

## Supplementary Material

Crystal structure: contains datablocks I, global. DOI: 10.1107/S1600536808040282/hy2169sup1.cif

Structure factors: contains datablocks I. DOI: 10.1107/S1600536808040282/hy2169Isup2.hkl

Additional supplementary materials:  crystallographic information; 3D view; checkCIF report

## Figures and Tables

**Table 1:** Selected bond lengths (Å)

Tl1—O3^i^	2.702 (5)
Tl1—O2	2.763 (5)
Tl1—O1*W*	2.882 (5)
Tl1—O4^ii^	2.952 (5)
Tl1—O1	3.135 (5)
Tl1—O1*W*^iii^	3.209 (5)
Tl1—O3^ii^	3.350 (5)

Symmetry codes: (i) 
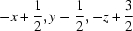
; (ii) 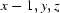; (iii) 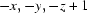.

**Table 2:** Hydrogen-bond geometry (Å, °)

*D*—H⋯*A*	*D*—H	H⋯*A*	*D*⋯*A*	*D*—H⋯*A*
N1—H4⋯O2^iv^	0.91	1.90	2.791 (8)	166
N1—H5⋯O3	0.91	1.85	2.741 (8)	166
N1—H6⋯O1^v^	0.91	2.11	2.828 (8)	136
N1—H6⋯O4^vi^	0.91	2.20	2.942 (8)	138
O1*W*—H7⋯O2^i^	0.85	2.06	2.909 (8)	172
O1*W*—H8⋯O1^v^	0.85	2.00	2.846 (8)	177
C3—H1⋯O1^v^	0.95	2.45	3.353 (9)	159
C6—H3⋯O3^vii^	0.99	2.59	3.523 (9)	157

Symmetry codes: (i) 
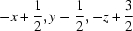
; (iv) 
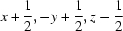
; (v) 
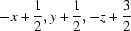
; (vi) 
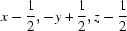
; (vii) 
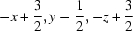
.

**Fig. 1.**
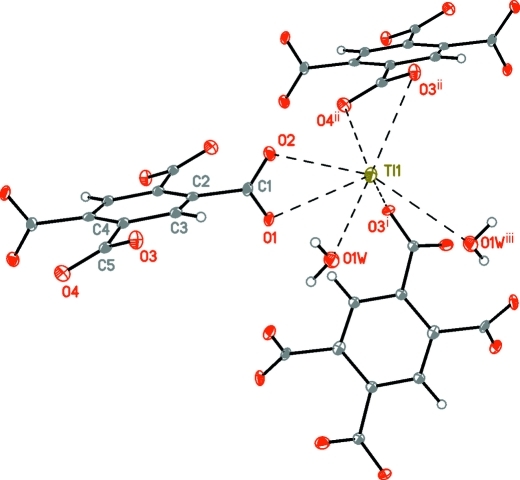
Coordination environment around the Tl atom in the title compound, showing an empty space on one side of the atom Tl1. Displacement ellipsoids are drawn at the 50% probability level. [Symmetry codes: (i) 1/2-x, -1/2+y, 3/2-z; (ii) -1+x, y, z; (iii) -x, -y, 1-z.]

**Fig. 2.**
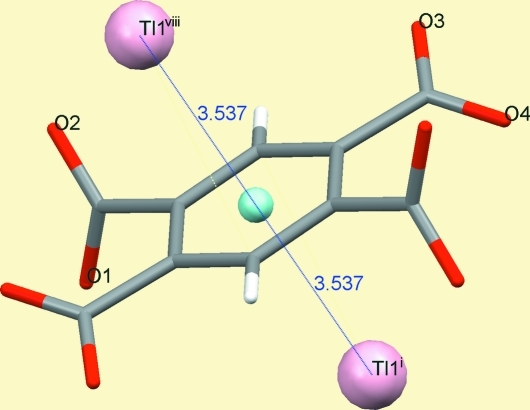
Tl···π interactions with a Tl–centroid distance of 3.537 (1) Å. [Symmetry codes: (i) 1/2-x, -1/2+y, 3/2-z; (viii) 1/2+x, 1/2-y, 1/2+z.]

## supplementary crystallographic information

### Comment

Thallium reagents, despite their inherent toxicity and cost, have played a conspicuous role in the development of modern inorganic and organometallic chemistry. Thallium(I) chemistry is very interesting due to a variety of reasons. (a) Thallium salts and complexes are often anhydrous. (b) The lone pair on thallium may or may not be stereochemically active. (c) High coordination number presents because of large size of Tl^I^ ion. (d) Thallium(I) complexes have potential ability to form metal–metal bonds and thallium(I) also forms complexes with aromatic hydrocarbons (Akhbari & Morsali, 2008).

The deprotonated forms of benzene-1,2,4,5-tetracarboxylic acid (btcH_4_) can act not only as hydrogen bond acceptors but also as hydrogen bond donors, depending on the deprotonated carboxylate groups, to give different supramolecular adducts (Fabelo *et al.*, 2005). There is an instance of benzene-1,2,4,5-tetracarboxylate coordinated to thallium in a mixed ligand system (Day & Luehrs, 1988). However, there are some coordination polymers reported that contain an anionic coordination polymer together with a cationic part, such as metal–organic framework-based hydrogen-bonded porous solids, [(pipzH_2_)M(btc)(H_2_O)_4_.4H_2_O]_n_ (M = Co^II^, Ni^II^, Zn^II^; pipz = piperazine) (Murugavel *et al.*, 2000). As the recent examples of this category, Cu^II^ and Zn^II^ anionic coordination polymers with ethylenediaminium and propane-1,2-diaminium (pn) as counter ions, {(enH_2_)[Cu(btc)].2.5H_2_O}_n_ (Rafizadeh *et al.*, 2007a) and {(pnH_2_)[Zn(btc)].4H_2_O]}_n_ (Rafizadeh *et al.*, 2007b), have been synthesized.

In the title compound (Fig. 1), the coordination behavior of carboxylate groups of btc are different. Compared with another Tl^I^ complex, [Tl(pydcH)]_n_ (pydcH_2_ = pyridine-2,6-dicarboxylic acid), with the bond lengths of Tl—O being 2.853 (6) and 3.019 (6) Å (Rafizadeh *et al.*, 2005), the Tl—O bond lengths of the title compound are in a more extended range [2.702 (5) to 3.350 (5) Å] (Table 1). In the crystal structure, O—H···O, N—H···O and C—H···O hydrogen bonds are present. Moreover, an interesting Tl···π interaction is found that is classified as cation···π interaction at a Tl–centroid distance of 3.537 (1) Å, as shown in Fig. 2. These interactions make all components assemble together in a packing arrangement.

As illustrated in Fig. 1, coordination number of the Tl^I^ atom is seven, with all coordinated atoms forced into one side of Tl^I^ and other side is left empty. This can be caused by the stereochemically active lone pair on Tl^I^ center. Based on crystal data available in the Cambridge Structural Database, stereochemistry of Pb^II^ complexes has been reviewed (Shimoni-Livny *et al.*, 1998). Evidently, in the case of Pb^II^ complexes when the lone pair appears to have no steric effects, the bonds with ligand donor atoms are arranged throughout the surface of encompassing sphere (holodirected coordination) and there are no marked differences in the Pb—*L* bond lengths. But the Pb^II^ complexes, in which the lone pair is stereochemically active, have hemidirected coordination and the Pb—*L* bonds are directed only to a part of the coordination sphere, leaving a gap in the distribution of bonds to the ligands. There are shorter Pb—*L* bonds away from the proposed site of the lone pair and longer Pb—*L* bonds adjacent to this site of the lone pair (Li *et al.*, 2008). Here also, the Tl^I^ atom shows the same behavior. In effect, the Tl1—O3^ii^ and Tl1—O1W^iii^ (symmetry codes: (ii) -1+x, y, z; (iii) -x, -y, 1-z), that are apparently longer than other bonds (see Fig. 1 and Table 1), lie on the side of the putative lone pair and the shorter bonds lie away from the site of the lone pair.

### Experimental

An aqueous solution of (enH_2_)_2_(btc).2H_2_O (0.34 g, 0.82 mmol), synthesized according to the literature (Rafizadeh *et al.*, 2006), was added dropwise to a solution of TlNO_3_ (0.061 g, 0.23 mmol) in water. The mixture was slightly heated and stirred for 5 h. The obtained clear solution with a volume of 40 ml was left at room temperature for 40 d. Then the lustrous pale yellow crystals were obtained (decomposing temperature > 673 K).

### Refinement

H atoms bound to C atoms were positioned geometrically and refined as riding atoms, with C—H = 0.95 (CH) and 0.99 (CH_2_) Å and N—H = 0.91 Å and with *U*_iso_(H) = 1.2*U*_eq_(C,N). H atoms of water molecule were located on a difference Fourier map and fixed in the refinements, with *U*_iso_(H) = 1.5*U*_eq_(O). The highest residual electron density was found 0.88 Å from atom Tl1 and the deepest hole 1.36 Å from atom Tl1.

### Crystal data

**Table:**

(C_2_H_10_N_2_)[Tl_2_(C_10_H_2_O_8_)(H_2_O)_2_)]	*F*(000) = 688
*M**_r_* = 757.01	*D*_x_ = 3.197 Mg m^−^^3^
Monoclinic, *P*2_1_/*n*	Mo *K*α radiation, λ = 0.71073 Å
Hall symbol: -P 2yn	Cell parameters from 1231 reflections
*a* = 9.925 (5) Å	θ = 3.4–32.7°
*b* = 7.073 (4) Å	µ = 20.53 mm^−^^1^
*c* = 11.325 (6) Å	*T* = 100 K
β = 98.397 (10)°	Prism, colourless
*V* = 786.5 (7) Å^3^	0.16 × 0.12 × 0.08 mm
*Z* = 2	

### Data collection

**Table:**

Bruker APEXII CCD area-detector diffractometer	1787 independent reflections
Radiation source: fine-focus sealed tube	1487 reflections with *I* > 2σ(*I*)
graphite	*R*_int_ = 0.061
φ and ω scans	θ_max_ = 27.5°, θ_min_ = 3.0°
Absorption correction: multi-scan (*SADABS*; Bruker, 2001)	*h* = −11→12
*T*_min_ = 0.064, *T*_max_ = 0.201	*k* = −9→9
5272 measured reflections	*l* = −14→14

### Refinement

**Table:**

Refinement on *F*^2^	Primary atom site location: structure-invariant direct methods
Least-squares matrix: full	Secondary atom site location: difference Fourier map
*R*[*F*^2^ > 2σ(*F*^2^)] = 0.031	Hydrogen site location: mixed
*wR*(*F*^2^) = 0.070	H-atom parameters constrained
*S* = 1.00	*w* = 1/[σ^2^(*F*_o_^2^) + (0.031*P*)^2^] where *P* = (*F*_o_^2^ + 2*F*_c_^2^)/3
1787 reflections	(Δ/σ)_max_ = 0.001
107 parameters	Δρ_max_ = 2.02 e Å^−^^3^
0 restraints	Δρ_min_ = −1.80 e Å^−^^3^

### Fractional atomic coordinates and isotropic or equivalent isotropic displacement parameters (Å^2^)

**Table:**

	*x*	*y*	*z*	*U*_iso_*/*U*_eq_	
Tl1	−0.05121 (3)	0.10847 (4)	0.68253 (2)	0.01266 (11)	
O1	0.1709 (5)	−0.1391 (7)	0.8360 (4)	0.0122 (11)	
O2	0.1667 (5)	0.1726 (8)	0.8595 (4)	0.0128 (10)	
O3	0.6799 (5)	0.2759 (7)	0.7972 (4)	0.0130 (11)	
O4	0.8315 (5)	0.0925 (8)	0.9073 (5)	0.0136 (11)	
C1	0.2231 (7)	0.0140 (11)	0.8761 (6)	0.0105 (14)	
C2	0.3648 (8)	0.0083 (10)	0.9445 (6)	0.0098 (14)	
C3	0.4680 (7)	0.0882 (10)	0.8919 (6)	0.0085 (8)	
H1	0.4463	0.1491	0.8168	0.010*	
C4	0.6041 (7)	0.0817 (10)	0.9465 (6)	0.0085 (8)	
C5	0.7143 (7)	0.1550 (10)	0.8785 (6)	0.0085 (8)	
C6	0.5359 (8)	0.0285 (11)	0.5608 (6)	0.0122 (15)	
H2	0.4801	−0.0087	0.6226	0.015*	
H3	0.6244	−0.0381	0.5775	0.015*	
N1	0.5585 (6)	0.2319 (8)	0.5655 (5)	0.0086 (12)	
H4	0.6070	0.2664	0.5067	0.010*	
H5	0.6058	0.2636	0.6378	0.010*	
H6	0.4768	0.2927	0.5550	0.010*	
O1W	0.1899 (5)	0.0253 (8)	0.5778 (5)	0.0167 (12)	
H7	0.2389	−0.0717	0.5965	0.025*	
H8	0.2341	0.1244	0.6018	0.025*	

### Atomic displacement parameters (Å^2^)

**Table:**

	*U*^11^	*U*^22^	*U*^33^	*U*^12^	*U*^13^	*U*^23^
Tl1	0.01330 (15)	0.01092 (15)	0.01267 (14)	−0.00149 (13)	−0.00175 (9)	0.00115 (12)
O1	0.011 (2)	0.010 (3)	0.014 (2)	−0.001 (2)	−0.0051 (19)	−0.002 (2)
O2	0.013 (3)	0.015 (3)	0.010 (2)	0.004 (2)	−0.001 (2)	0.002 (2)
O3	0.014 (3)	0.012 (3)	0.012 (2)	−0.001 (2)	0.000 (2)	0.006 (2)
O4	0.014 (3)	0.013 (3)	0.015 (2)	0.000 (2)	0.003 (2)	0.003 (2)
C1	0.010 (3)	0.017 (4)	0.005 (3)	0.001 (3)	0.000 (3)	0.001 (3)
C2	0.015 (4)	0.004 (3)	0.010 (3)	0.003 (3)	0.000 (3)	−0.002 (3)
C3	0.011 (2)	0.005 (2)	0.0092 (17)	−0.0004 (15)	0.0007 (15)	−0.0011 (15)
C4	0.011 (2)	0.005 (2)	0.0092 (17)	−0.0004 (15)	0.0007 (15)	−0.0011 (15)
C5	0.011 (2)	0.005 (2)	0.0092 (17)	−0.0004 (15)	0.0007 (15)	−0.0011 (15)
C6	0.014 (4)	0.007 (3)	0.014 (4)	0.001 (3)	−0.002 (3)	0.001 (3)
N1	0.008 (3)	0.008 (3)	0.009 (3)	0.001 (2)	−0.002 (2)	0.003 (2)
O1W	0.019 (3)	0.013 (3)	0.018 (3)	0.002 (2)	−0.001 (2)	0.001 (2)

### Geometric parameters (Å, °)

**Table:**

Tl1—O3^i^	2.702 (5)	C3—C4	1.402 (10)
Tl1—O2	2.763 (5)	C3—H1	0.9500
Tl1—O1W	2.882 (5)	C4—C2^iv^	1.384 (10)
Tl1—O4^ii^	2.952 (5)	C4—C5	1.518 (10)
Tl1—O1	3.135 (5)	C6—N1	1.456 (10)
Tl1—O1W^iii^	3.209 (5)	C6—C6^v^	1.510 (14)
Tl1—O3^ii^	3.350 (5)	C6—H2	0.9900
O1—C1	1.257 (9)	C6—H3	0.9900
O2—C1	1.256 (9)	N1—H4	0.9100
O3—C5	1.266 (8)	N1—H5	0.9100
O4—C5	1.242 (9)	N1—H6	0.9100
C1—C2	1.503 (10)	O1W—H7	0.8500
C2—C3	1.378 (10)	O1W—H8	0.8500
C2—C4^iv^	1.384 (10)		
			
O3^i^—Tl1—O2	114.26 (16)	C4—C3—H1	119.2
O3^i^—Tl1—O1W	106.77 (16)	C2^iv^—C4—C3	118.9 (6)
O2—Tl1—O1W	73.92 (15)	C2^iv^—C4—C5	121.8 (6)
O3^i^—Tl1—O4^ii^	69.06 (15)	C3—C4—C5	119.0 (6)
O2—Tl1—O4^ii^	75.31 (15)	O4—C5—O3	125.0 (6)
O1W—Tl1—O4^ii^	143.40 (15)	O4—C5—C4	117.5 (6)
O3^i^—Tl1—O1	76.67 (15)	O3—C5—C4	117.5 (6)
O2—Tl1—O1	43.70 (14)	N1—C6—C6^v^	110.3 (8)
O1W—Tl1—O1	63.61 (15)	N1—C6—H2	109.6
O4^ii^—Tl1—O1	80.47 (14)	C6^v^—C6—H2	109.6
C1—O1—Tl1	86.5 (4)	N1—C6—H3	109.6
C1—O2—Tl1	104.3 (4)	C6^v^—C6—H3	109.6
C5—O3—Tl1^vi^	127.4 (4)	H2—C6—H3	108.1
C5—O4—Tl1^vii^	103.4 (4)	C6—N1—H4	109.5
O2—C1—O1	124.3 (6)	C6—N1—H5	109.5
O2—C1—C2	117.7 (7)	H4—N1—H5	109.5
O1—C1—C2	118.0 (7)	C6—N1—H6	109.5
C3—C2—C4^iv^	119.4 (7)	H4—N1—H6	109.5
C3—C2—C1	117.7 (6)	H5—N1—H6	109.5
C4^iv^—C2—C1	122.8 (6)	Tl1—O1W—H7	122.8
C2—C3—C4	121.6 (7)	Tl1—O1W—H8	96.9
C2—C3—H1	119.2	H7—O1W—H8	109.6
			
O3^i^—Tl1—O1—C1	−155.0 (4)	O2—C1—C2—C4^iv^	116.3 (8)
O2—Tl1—O1—C1	−5.8 (4)	O1—C1—C2—C4^iv^	−66.1 (9)
O1W—Tl1—O1—C1	88.3 (4)	C4^iv^—C2—C3—C4	−0.4 (11)
O4^ii^—Tl1—O1—C1	−84.5 (4)	C1—C2—C3—C4	−177.0 (6)
O3^i^—Tl1—O2—C1	39.1 (4)	C2—C3—C4—C2^iv^	0.4 (11)
O1W—Tl1—O2—C1	−62.4 (4)	C2—C3—C4—C5	174.0 (6)
O4^ii^—Tl1—O2—C1	97.5 (4)	Tl1^vii^—O4—C5—O3	−30.4 (8)
O1—Tl1—O2—C1	6.0 (4)	Tl1^vii^—O4—C5—C4	150.1 (5)
Tl1—O2—C1—O1	−12.6 (8)	Tl1^vi^—O3—C5—O4	−125.2 (6)
Tl1—O2—C1—C2	164.9 (5)	Tl1^vi^—O3—C5—C4	54.4 (8)
Tl1—O1—C1—O2	10.7 (6)	C2^iv^—C4—C5—O4	17.5 (10)
Tl1—O1—C1—C2	−166.7 (6)	C3—C4—C5—O4	−156.0 (6)
O2—C1—C2—C3	−67.2 (8)	C2^iv^—C4—C5—O3	−162.1 (7)
O1—C1—C2—C3	110.4 (8)	C3—C4—C5—O3	24.5 (10)

Symmetry codes: (i) −*x*+1/2, *y*−1/2, −*z*+3/2; (ii) *x*−1, *y*, *z*; (iii) −*x*, −*y*, −*z*+1; (iv) −*x*+1, −*y*, −*z*+2; (v) −*x*+1, −*y*, −*z*+1; (vi) −*x*+1/2, *y*+1/2, −*z*+3/2; (vii) *x*+1, *y*, *z*.

### Hydrogen-bond geometry (Å, °)

**Table:**

*D*—H···*A*	*D*—H	H···*A*	*D*···*A*	*D*—H···*A*
N1—H4···O2^viii^	0.91	1.90	2.791 (8)	166
N1—H5···O3	0.91	1.85	2.741 (8)	166
N1—H6···O1^vi^	0.91	2.11	2.828 (8)	136
N1—H6···O4^ix^	0.91	2.20	2.942 (8)	138
O1W—H7···O2^i^	0.85	2.06	2.909 (8)	172
O1W—H8···O1^vi^	0.85	2.00	2.846 (8)	177
C3—H1···O1^vi^	0.95	2.45	3.353 (9)	159
C6—H3···O3^x^	0.99	2.59	3.523 (9)	157

Symmetry codes: (viii) *x*+1/2, −*y*+1/2, *z*−1/2;  (vi) −*x*+1/2, *y*+1/2, −*z*+3/2; (ix) *x*−1/2, −*y*+1/2, *z*−1/2; (i) −*x*+1/2, *y*−1/2, −*z*+3/2; (x) −*x*+3/2, *y*−1/2, −*z*+3/2.
